# VCF/Plotein: visualization and prioritization of genomic variants from human exome sequencing projects

**DOI:** 10.1093/bioinformatics/btz458

**Published:** 2019-06-04

**Authors:** Raul Ossio, O Isaac Garcia-Salinas, Diego Said Anaya-Mancilla, Jair S Garcia-Sotelo, Luis A Aguilar, David J Adams, Carla Daniela Robles-Espinoza

**Affiliations:** 1 Laboratorio Internacional de Investigación sobre el Genoma Humano, Universidad Nacional Autónoma de México, Querétaro 76230, Mexico; 2 Experimental Cancer Genetics, Wellcome Sanger Institute, Hinxton, Cambridge CB10 1SA, UK

## Abstract

**Motivation:**

Identifying disease-causing variants from exome sequencing projects remains a challenging task that often requires bioinformatics expertise. Here we describe a user-friendly graphical application that allows medical professionals and bench biologists to prioritize and visualize genetic variants from human exome sequencing data.

**Results:**

We have implemented VCF/Plotein, a graphical, fully interactive web application able to display exome sequencing data in VCF format. Gene and variant information is extracted from Ensembl. Cross-referencing with external databases and application-based gene and variant filtering have also been implemented. All data processing is done locally by the user’s CPU to ensure the security of patient data.

**Availability and implementation:**

Freely available on the web at https://vcfplotein.liigh.unam.mx. Website implemented in JavaScript using the Vue.js framework, with all major browsers supported. Source code freely available for download at https://github.com/raulossio/VCF-plotein.

**Supplementary information:**

Supplementary data are available at *Bioinformatics* online.

## 1 Introduction

Exome sequencing (ES) has been highly successful at identifying genetic variation contributing to a large number of human phenotypes and diseases (Do *et al.*, 2012; Gilissen *et al.*, 2011). However, the actual process of identifying disease-causing variants and mutations remains a challenging task, and often one that requires at least some bioinformatics knowledge. This is due mainly to the sheer number of variants routinely identified in ES projects, the diversity of biological mechanisms by which variants may act, and the need to integrate large amounts of information from both pathogenicity scoring algorithms and clinical and population databases.

In this context, several software tools have been developed that are able to filter, display and contextualize exome sequencing data in order to accelerate the discovery of disease-causing variants. However, these platforms either require a good understanding of the command line (Paila *et al.*, 2013), have an interactive web interface but do not leverage external gene annotations that enrich biological interpretation (Hart *et al.*, 2016; Salatino and Ramraj, 2017), or do not support variant visualization at the protein level (Alemán *et al.*, 2014; Salatino and Ramraj, 2017).

Here, we introduce VCF/Plotein, a user-friendly graphical web application to both visualize and prioritize variants from exome sequencing studies that requires minimal bioinformatics knowledge. As such, this application can be used equally by bioinformaticians, by biologists whose projects involve exome sequencing, or by medical professionals studying a particular disease or gene.

## 2 Materials and methods

VCF/Plotein has been implemented entirely as a single-page application hosted on a server with a 2-core Intel Xeon E5-4627 v4 2.60 Ghz processor running a VMware 6.5.0 virtual machine over a Linux Centos 7.5 operating system. The server also has 4 GB of RAM and a solid-state hard disk drive with 1 TB of storage space. The application has been written mainly in JavaScript and uses the Vue.js-based Nuxt.js framework to control the storage, flow and presentation of information in the browser. A purpose-made API has been developed to obtain information from locally-installed external databases [gnomAD (version: 2.1 size: 59.23 GB) (Lek *et al.*, 2016), dbSNP (build: 151, size: 14.6 GB) (Sherry *et al.*, 2001), COSMIC (version: 86, size: 421.8 MB) (Forbes *et al.*, 2017), ClinVar (version: 86, size: 170.7 MB) (Landrum *et al.*, 2014), phenotype relationships from the Human Phenotype Ontology database (version: February 2019, size: 5.9 mb) (Kohler *et al.* 2019) and GO term information (version: September 2018, size: 7mb) (Ashburner *et al.*, 2000) for each annotated gene]. VCF/Plotein works with files in the variant call format (VCF) (Danecek *et al.*, 2011). Upon loading, a VCF file is validated and, after identifying the assembly version from the appropriate line, genes with variants are quickly found by matching an interval tree algorithm to the internal coordinate indexes containing each gene’s genomic positions. This generates a list with all the genes represented in the VCF, which can be filtered in different ways. Once a gene is selected, information about protein-coding transcripts and functional domains is extracted from Ensembl via the REST API (Zerbino *et al.*, 2018). Consequences from all variants falling within the selected gene, as well as their pathogenicity scores by SIFT (Ng and Henikoff, 2003) and PolyPhen (Adzhubei *et al.*, 2010, 2013), are obtained via the Ensembl Variant Effect Predictor (McLaren *et al.*, 2016). Cross-referencing with supported external databases is then performed by querying our internal database using the Elasticsearch search engine (Supplementary Fig. S1). All collected information is stored as a collection of objects in JSON format, returned to the web browser and depicted over a customizable plot of the primary structure of the canonical transcript made using the D3.js library (Supplementary Fig. S2). All operations, except for the search of naked genomic positions in supported external databases, are performed locally by the user’s CPU.

## 3 Results

### 3.1 Overview

The only requirements to run VCF/Plotein are a computer with an internet connection and a VCF file. Once the user loads the VCF file, the genome assembly is identified, genes with variants are found, and a list of criteria is displayed to aid with gene prioritization (Supplementary Fig. S3). Once a gene is selected, a new page is shown with the primary protein structure of its canonical transcript with its domains and other features along with all its recorded variants. Variants are shown with an indication of their frequency among samples in the VCF file, their transcript consequences, and their presence or absence in the gnomAD, dbSNP, ClinVar and COSMIC databases (Supplementary Fig. S2). The user can click on any variant to access further information about it, such as its genomic coordinates, a prediction of its pathogenicity according to SIFT and PolyPhen, and a list of carrier samples. The left-hand menu allows the user to load a new VCF file, to select a different gene, to select a different transcript, to select which protein domains and features to show, to filter variants, to analyze sample IDs, and finally to bookmark the selected features. Using the top menu, variant information can also be displayed and downloaded in table format, which includes zygosity information for each carrier sample, as well as printed in the SVG vector image file format or the PNG raster graphics format.

### 3.2 Data security

The API and the internal databases have been installed behind a Fortinet firewall, and run over an HTTPS port with a SSL certificate for secure data transfer. No sensitive sample information is uploaded to the server. Sensitive data comprise the name or ID of the samples, sample genotype information, any annotation previously added to the VCF file by the user, or information in the VCF headers. The only information sent to the servers is naked genomic positions (chromosome, position and base change), in order to retrieve any relevant information present in public databases. Therefore, the server does not hold or save any sample information, an important feature given the data security policy that many patient-focused sequencing projects are bound by. All data processing, including construction of the JSON object and graphing of primary protein structures, is done locally on the user’s computer.

### 3.3 Variant filtering and visualization

Variants falling in any selected protein-coding transcript from any gene can be filtered and plotted. Users can filter variants by protein consequence, by clinical prediction, by pathogenicity score or by their allelic frequency in the gnomAD database, or can select a custom subset to display. Users can also select which protein domains and features to plot. The customized protein plot can then be exported as an SVG or PNG file.

### 3.4 Performance

VCF/Plotein is able to process VCF files from exome sequencing studies in a reduced time frame. One of the key aspects regarding performance has to do with the opening and loading of the VCF file, which requires as much RAM as the size of the file. Therefore, there is no hard limit in this step: Computers with more RAM will perform better at this task and will be able to open bigger files. A similar relationship exists between processor type and processing time: Processors with faster clock speeds will read the VCF file information faster. Since the application is run in the browser, the operating system does not play an important role in the performance of the application. Other time-consuming steps are those that require sending and receiving data over the internet, which are affected by connection data transfer speeds and the number of variants sent to the servers for querying the databases. To illustrate the performance of VCF/Plotein under different system architectures, processor types and memory characteristics, we have tested our application with different file sizes on a number of different machines (Supplementary Table S1). Although VCF/Plotein should run without issues on the majority of web browsers, it has been tested on the Chrome browser in the MacOS and Linux operating systems, as well as on the Edge browser in the Windows 10 operating system.

### 3.5 Bookmarks

Bookmarks allow users to easily save any selected features from any number of gene transcripts in a text file (in JSON format) which can subsequently be loaded into VCF/Plotein.

### 3.6 Comparison with other similar tools

Other available software tools perform some of the functions of VCF/Plotein, but either require at least some bioinformatics expertise, do not leverage information from external databases, do not allow users to visualize their own exome data, or are not freely available (Supplementary Table S2).

## 4 Use case: finding pathogenic variants in the *BAP1* gene

To illustrate how to use VCF/Plotein, we have provided a use case based on a real VCF file from O’Shea *et al.* (2017), who performed functional studies to identify those variants in the *BAP1* gene likely to confer a higher risk of melanoma. We have supplemented this VCF file with simulated mutation data to add information from more genes. In the accompanying Supplementary Text, available in the Online Materials, we go through the typical filtering steps a researcher may follow to prioritize variants within this gene, which yields 4 variants, three of which were found to be functional in the original publication.

## 5 Discussion

We anticipate that VCF/Plotein will allow researchers, especially in small labs, to focus on biologically relevant questions instead of having to learn to install software dependencies, learn to use variant-annotation and cross-referencing tools, and become familiar with the UNIX and/or the MySQL command line. The main advantages that this tool provides over other similar software are its ease of use, the ability to display information from a custom VCF file, that it is freely available, and that it can process files locally. We have illustrated with a use case that, by applying a number of filters, a researcher can identify a small subset of variants within a gene that contains those found to be deleterious to gene function. By combining variant filtering and annotation in a single graphical and interactive tool, we have shown that variant prioritization and visualization become easier, faster and more intuitive.

## Supplementary Material

btz458_Supplementary_Materials

## References

[btz458-B1] Adzhubei I. et al (2013) Predicting functional effect of human missense mutations using PolyPhen-2. Curr. Protoc. Hum. Genet., Chapter 7, Unit7.20. doi: 10.1002/0471142905.hg0720s76 10.1002/0471142905.hg0720s76 PMC4480630 23315928

[btz458-B2] Adzhubei I.A. et al (2010) A method and server for predicting damaging missense mutations. Nat. Methods, 7, 248–249. 20354512 10.1038/nmeth0410-248 PMC2855889

[btz458-B3] Alemán A. et al (2014) A web-based interactive framework to assist in the prioritization of disease candidate genes in whole-exome sequencing studies. Nucleic Acids Res., 42, W88–93. 24803668 10.1093/nar/gku407 PMC4086071

[btz458-B4] Ashburner M. et al (2000) Gene ontology: tool for the unification of biology. The Gene Ontology Consortium. Nat. Genet., 25, 25–29. 10802651 10.1038/75556 PMC3037419

[btz458-B5] Danecek P. et al (2011) The variant call format and VCFtools. Bioinformatics, 27, 2156–2158. 21653522 10.1093/bioinformatics/btr330 PMC3137218

[btz458-B6] Do R. et al (2012) Exome sequencing and complex disease: practical aspects of rare variant association studies. Hum. Mol. Genet., 21, R1–9. 22983955 10.1093/hmg/dds387 PMC3459641

[btz458-B7] Forbes S.A. et al (2017) COSMIC: somatic cancer genetics at high-resolution. Nucleic Acids Res., 45, D777–D783. 27899578 10.1093/nar/gkw1121 PMC5210583

[btz458-B8] Gilissen C. et al (2011) Unlocking Mendelian disease using exome sequencing. Genome Biol., 12, 228. 21920049 10.1186/gb-2011-12-9-228 PMC3308044

[btz458-B9] Hart S.N. et al (2016) VCF-Miner: GUI-based application for mining variants and annotations stored in VCF files. Brief. Bioinform., 17, 346–351. 26210358 10.1093/bib/bbv051 PMC4793895

[btz458-B10] Kohler S. et al (2019) Expansion of the Human Phenotype Ontology (HPO) knowledge base and resources. Nucleic Acids Res., 47, D1018–D1027. 30476213 10.1093/nar/gky1105 PMC6324074

[btz458-B11] Landrum M.J. et al (2014) ClinVar: public archive of relationships among sequence variation and human phenotype. Ncucleic Acids Res., 42, D980–D985. 10.1093/nar/gkt1113 PMC3965032 24234437

[btz458-B12] Lek M. et al (2016) Analysis of protein-coding genetic variation in 60,706 humans. Nature, 536, 285–291. 27535533 10.1038/nature19057 PMC5018207

[btz458-B13] McLaren W. et al (2016) The Ensembl variant effect predictor. Genome Biol., 17, 122. 27268795 10.1186/s13059-016-0974-4 PMC4893825

[btz458-B14] Ng P.C., Henikoff S. (2003) SIFT: predicting amino acid changes that affect protein function. Nucleic Acids Res., 31, 3812–3814. 12824425 10.1093/nar/gkg509 PMC168916

[btz458-B15] O’Shea S.J. et al (2017) A population-based analysis of germline BAP1 mutations in melanoma. Hum. Mol. Genet., 26, 717–728. 28062663 10.1093/hmg/ddw403 PMC5409081

[btz458-B16] Paila U. et al (2013) GEMINI: integrative exploration of genetic variation and genome annotations. PLoS Comput. Biol., 9, e1003153. 23874191 10.1371/journal.pcbi.1003153 PMC3715403

[btz458-B17] Salatino S., Ramraj V. (2017) BrowseVCF: a web-based application and workflow to quickly prioritize disease-causative variants in VCF files. Brief. Bioinform., 18, 774–779. 27373737 10.1093/bib/bbw054 PMC5862253

[btz458-B18] Sherry S.T. et al (2001) dbSNP: the NCBI database of genetic variation. Nucleic Acids Res., 29, 308–311. 11125122 10.1093/nar/29.1.308 PMC29783

[btz458-B19] Zerbino D.R. et al (2018) Ensembl 2018. Nucleic Acids Res., 46, D754–D761. 29155950 10.1093/nar/gkx1098 PMC5753206

