# Supplementary material for: VCF/Plotein: visualization and prioritization of genomic variants from human exome sequencing projects
Source: Bioinformatics. 2019 Jun 4;35(22):4803–5. doi: 10.1093/bioinformatics/btz458 (PMC6853650; doi:10.1093/bioinformatics/btz458)
Supplement: btz458_Supplementary_Materials [file btz458_supplementary_materials.zip › btz458-suppl_data/Supplementary_Legends.docx]

**Supplementary Figure Legends**

**Supplementary Figure 1. Chart depicting the VCF/Plotein process flow and system architecture**. The colours of each box in the top panel correspond to the colour of the system architecture element in which it is executed (in the bottom panel), e.g., actions in green boxes are executed by the user, those in blue boxes are executed by the user’s browser, and actions in red, purple and orange are processes used to obtain information from diverse databases (each color represents a different database). The numbers in the figure indicate the process flow, and the same actions in a cartoon of the system architecture:

1. The user selects and loads the VCF file (compressed or uncompressed) in the Open file panel of the web app.
2. The program opens the file (and decompresses it, if it is in the vcf.gz format) and parses it.
3. The genes represented in the VCF are found by introducing the variants in the file to an interval tree algorithm.
4. The list of genes is sent to the server to generate the list of GO terms and phenotypes related to the genes represented in the VCF file.
5. The GO terms and phenotype filters are created on the web app.
6. The user selects the gene of interest to generate its graph.
7. The program extracts all the variants of the selected gene and then classifies their associated information into non-sensitive and sensitive data. Sensitive data is defined as sample names or IDs, sample genotype information, any annotation previously added to the VCF file by the user, or information in the VCF’s file headers. Non-sensitive data are chromosome, position and nucleotide base change. Non-sensitive data are sent to the Ensembl REST API to get Variant Effect Predictor information. The program also sends these non-sensitive data to the servers at the National Autonomous University of Mexico to retrieve information from public databases (gnomAD, dbSNP, ClinVar and COSMIC) using the Elasticsearch search engine.
8. At the same time that the non-sensitive variant information is sent to the servers, the program starts to process the information about the samples locally on the user’s computer.
9. After the program has all the information it needs from Ensembl and the different databases, it processes it and displays it in a graph of the primary protein structure.
10. Finally, the user is able to interact fully with the application, accessing variant-level information and selecting various filters.

**Supplementary Figure 2. VCF/Plotein working screen**. Screen depicting protein-level information for the selected gene. 1) The primary protein structure of the canonical transcript is shown alongside protein domains and other features with the the genetic variants shown as lollipops. The colours of these “lollipops” indicate the protein consequence, and the squares below indicate the presence or absence of the variant in different external databases. 2) The top panel shows information about the gene and the transcript being visualised, as well as either general information about all variants in the gene or, if any variant is selected, information specific to it (genomic location, protein consequences and carrier samples). 3) If a particular variant is selected, the bottom panel shows its SIFT, PolyPhen and MaxEntScan scores (if available), and information about its presence in other databases (dbSNP, ClinVar, COSMIC, gnomAD). 4) The left-hand side menu allows users to customise and interact with the plot, allowing them to upload a different file, or select a different gene in the current file or transcript, display different protein domains, and filter variants in various ways. 5) The top menu allows the user to visualise and export information in different formats.

**Supplementary Figure 3. Loading and data filtering screen.** 1) A user can click in the gray box to upload a VCF file or a bookmark (created with VCF/Plotein from a previously loaded file). The reference genome assembly is automatically identified from the appropriate line in the file. 2) A list of different criteria to aid with gene prioritisation is shown: Users can select genes based on a preferred chromosome, biological process, molecular function, cellular component of associated phenotype. 3) A list of genes matching the selected criteria is shown, from which then one can be selected to display in the protein-level information screen.

**Supplementary Table Legends**

**Supplementary Table 1. Performance of VCF/Plotein after loading three different VCF files in different operating systems with a range of hardware specifications**. Tests were performed in the Google Chrome browser on MacOS and Linux operating systems, as well as in the Edge browser on Windows 10. All times are in milliseconds (ms).

**Supplementary Table 2. Comparison of the main features of VCF/Plotein with those of other similar tools**. GEMINI, VCF-miner, BrowseVCF, BIERapp, exomeSuite, PeCan, VCF iobio, VizGVar, VariantStudio*, Alamut*, Ingenuity*, VarSeq* and IGV. *These tools are not freely available, we have obtained their characteristics from their available documentation. **Program allows to select variants by consequence type but not by allele frequency or pathogenicity prediction.

**Supplementary References**

[Alemán, Alejandro, Francisco Garcia-Garcia, Francisco Salavert, Ignacio Medina, and Joaquín Dopazo. 2014. “A Web-Based Interactive Framework to Assist in the Prioritization of Disease Candidate Genes in Whole-Exome Sequencing Studies.” Nucleic Acids Research 42 (Web Server issue): W88–93.](http://paperpile.com/b/5uHkux/cNF4)

[Hart, Steven N., Patrick Duffy, Daniel J. Quest, Asif Hossain, Mike A. Meiners, and Jean-Pierre Kocher. 2016. “VCF-Miner: GUI-Based Application for Mining Variants and Annotations Stored in VCF Files.” Briefings in Bioinformatics 17 (2): 346–51.](http://paperpile.com/b/5uHkux/kOAO)

[“Ingenuity Variant Analysis.” n.d. Accessed March 28, 2019.](http://paperpile.com/b/5uHkux/HOiN) <https://www.qiagenbioinformatics.com/products/ingenuity-variant-analysis/>[.](http://paperpile.com/b/5uHkux/HOiN)

[Maranhao, B., P. Biswas, J. L. Duncan, K. E. Branham, G. A. Silva, M. A. Naeem, S. N. Khan, et al. 2014. “exomeSuite: Whole Exome Sequence Variant Filtering Tool for Rapid Identification of Putative Disease Causing SNVs/indels.” Genomics 103 (2-3): 169–76.](http://paperpile.com/b/5uHkux/HfUf)

[Miller, Chase A., Yi Qiao, Tonya DiSera, Brian D’Astous, and Gabor T. Marth. 2014. “Bam.iobio: A Web-Based, Real-Time, Sequence Alignment File Inspector.” Nature Methods 11 (12): 1189.](http://paperpile.com/b/5uHkux/0rRe)

[Paila, Umadevi, Brad A. Chapman, Rory Kirchner, and Aaron R. Quinlan. 2013. “GEMINI: Integrative Exploration of Genetic Variation and Genome Annotations.” PLoS Computational Biology 9 (7): e1003153.](http://paperpile.com/b/5uHkux/mKSh)

[Salatino, Silvia, and Varun Ramraj. 2017. “BrowseVCF: A Web-Based Application and Workflow to Quickly Prioritize Disease-Causative Variants in VCF Files.” Briefings in Bioinformatics 18 (5): 774–79.](http://paperpile.com/b/5uHkux/5yoV)

[Solano-Román, Antonio, Verónica Alfaro-Arias, Carlos Cruz-Castillo, and Allan Orozco-Solano. 2018. “Visualization Portal for Genetic Variation (VizGVar): A Tool for Interactive Visualization of SNPs and Somatic Mutations in Exons, Genes and Protein Domains.” Bioinformatics  34 (6): 1048–49.](http://paperpile.com/b/5uHkux/u1ki)

[“The Alamut Software Suite.” n.d. Accessed March 22, 2019.](http://paperpile.com/b/5uHkux/76q7) <https://www.interactive-biosoftware.com/products/>[.](http://paperpile.com/b/5uHkux/76q7)

[Thorvaldsdóttir, Helga, James T. Robinson, and Jill P. Mesirov. 2013. “Integrative Genomics Viewer (IGV): High-Performance Genomics Data Visualization and Exploration.” Briefings in Bioinformatics 14 (2): 178–92.](http://paperpile.com/b/5uHkux/cl9Z)

[“VariantStudio.” n.d. Accessed April 3, 2019.](http://paperpile.com/b/5uHkux/ZysN) <https://support.illumina.com/sequencing/sequencing_software/variantstudio.html>[.](http://paperpile.com/b/5uHkux/ZysN)

[“VarSeq.” n.d.](http://paperpile.com/b/5uHkux/OcGe) <http://goldenhelix.com/products/VarSeq/index.html>[.](http://paperpile.com/b/5uHkux/OcGe)

[Zhou, Xin, Michael N. Edmonson, Mark R. Wilkinson, Aman Patel, Gang Wu, Yu Liu, Yongjin Li, et al. 2016. “Exploring Genomic Alteration in Pediatric Cancer Using ProteinPaint.” Nature Genetics 48 (1): 4–6.](http://paperpile.com/b/5uHkux/SmU9)
