# Supplementary material for: VCF/Plotein: visualization and prioritization of genomic variants from human exome sequencing projects
Source: Bioinformatics. 2019 Jun 4;35(22):4803–5. doi: 10.1093/bioinformatics/btz458 (PMC6853650; doi:10.1093/bioinformatics/btz458)

**Use case**: Using VCF/Plotein to perform variant prioritisation

Based on data from O’Shea *et al* (2017) *Hum Mol Genet* **26**(4):717-728

Prioritising genetic variants in the *BAP1* gene that may predispose to melanoma

In this project, we have sequenced all exons of a few genes in 1,977 melanoma cases and 754 controls ascertained in the same hospital (St James’s Hospital, Leeds), who are all of European ancestry. We know that some rare germline variants of the *BAP1* gene have been associated to melanocytic lesions and indeed to a range of other cancers, so it would be beneficial to the medical community if we were able to prioritise variants out of all those found in a large sequencing effort for functional testing and other clinical evaluations. This would allow us to gather enough knowledge for their inclusion in genetic testing and patient counselling.

The sequence information for all 1,977 cases and 754 controls is stored in a VCF file (*use_case.vcf.gz*, provided as a downloadable file in the **Online Materials**), which we load into VCF/Plotein (1):


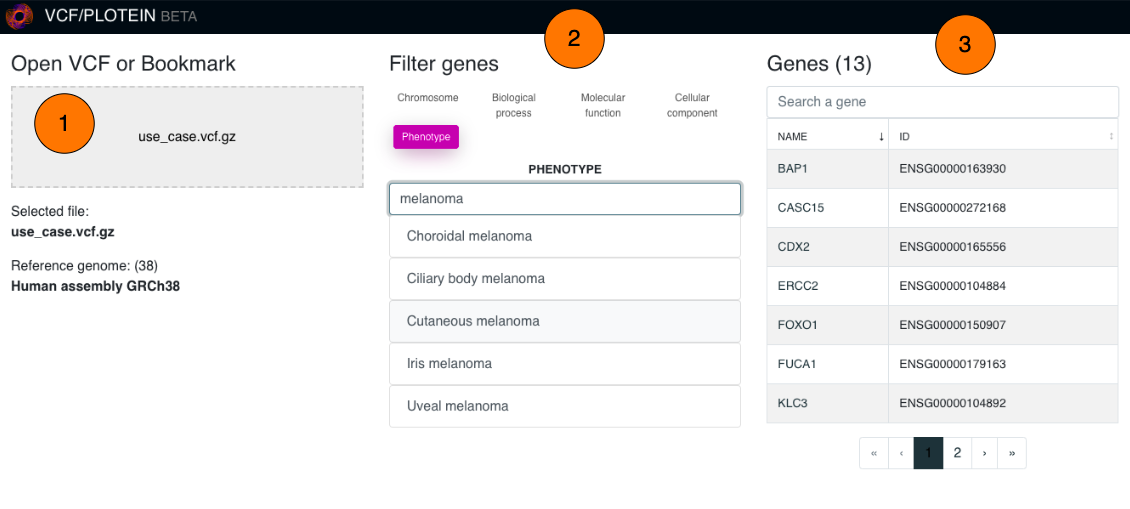


From here we can see that we have information for these few genes. We can look for genes associated to melanoma by selecting “Cutaneous melanoma” in the Phenotype filter (2) or by directly writing a query gene into the search box (3). We click on *BAP1*.

We select the transcript of interest from the
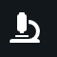
 menu, which is *ENST00000460680* (the longest transcript). **Note:** VCF/Plotein displays the canonical transcript, as per information in the Ensembl database, by default.


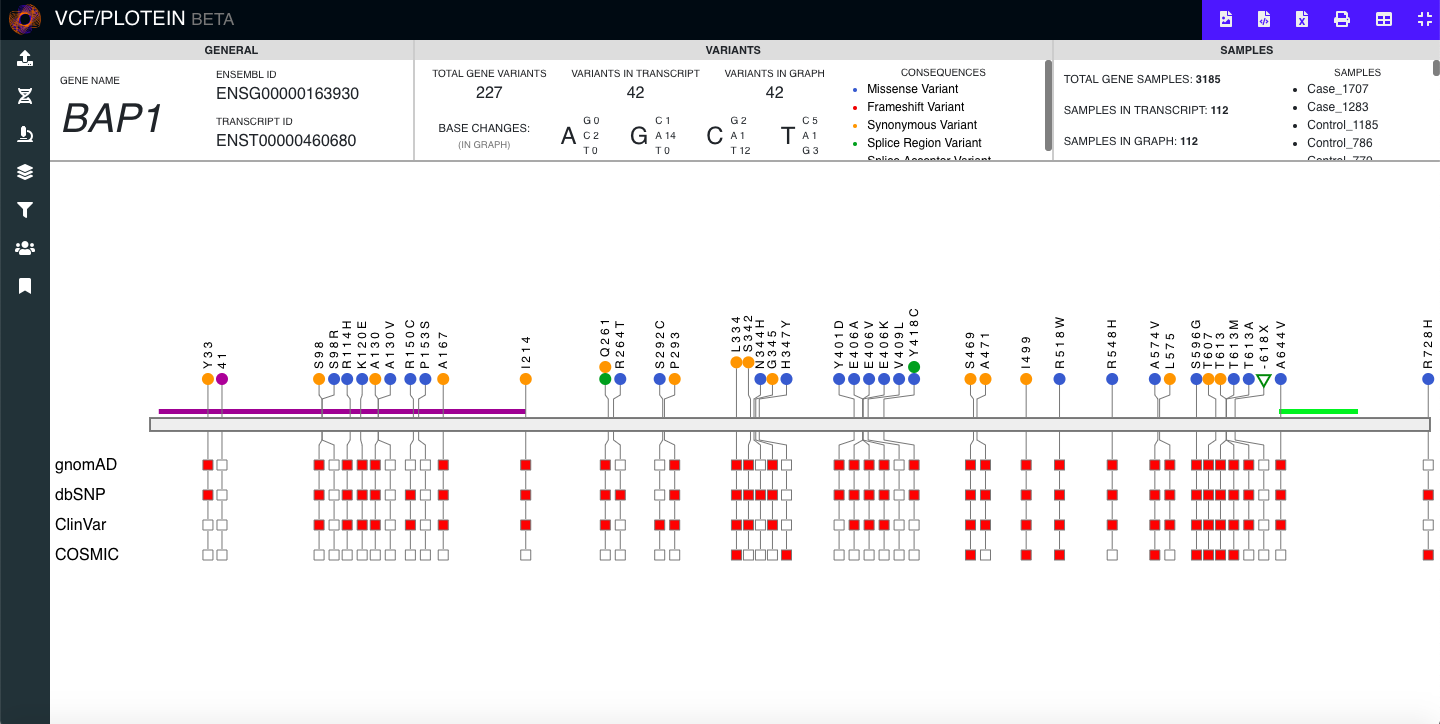


Here, we can see a graph of all variants found across all individuals in the VCF. If we click through the variants, we get more information about carrier samples, pathogenicity predictions, MAxEntScan scores when available and presence or absence of variants in diverse databases.

In order to do a first possible filter of our variants, we can focus on those that are predicted to be most deleterious to the protein (typically, splice acceptor or donor variants, stops gained, or frameshift variants). A quick look in the
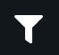
 menu shows us that we have a frameshift variant (at amino acid 618) and a splice acceptor (near amino acid 4). Therefore, these become interesting variants for functional follow-up.

But what about other variants? These may change the protein structure but their effects to the protein may be a bit more difficult to predict. This is why it is useful to integrate several lines of evidence when making this prioritisation.

The first thing we may do is focus on variants falling in important protein domains, as these may be the ones affecting important protein functions. In our example, there is one PFam domain in the N-terminal part of the protein marked as ‘Peptidase C12, ubiquitin carboxyl-terminal hydrolase’. *BAP1* primarily functions as a deubiquitinase, so this seems like a plausible region of the protein to focus on. **Note:** VCF/Plotein displays all domains annotated in the PFam database, as per information in the Ensembl database, by default.

From here, if we display all variant consequences, we can see that we have 11 potential variants (excluding the splice acceptor we have seen already) falling into this functional domain.

We can then display only those variants that change the protein structure (i.e., non-silent variants). In order to do this, we can go to the menu
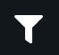
 and filter by consequence, selecting only those variants that are missense:

By doing this, we see that there are only 6 remaining variants, all except P153S falling in cases (something we can see by looking at the header when clicking on each variant or by filtering to show only cases in the left menu). A common step that follows in prioritisation exercises is to only focus on variants that are scored “deleterious” and “probably damaging” by SIFT and PolyPhen-2 respectively, and that are very rare in the general population. Applying all these filters then leaves us with only two variants, S98R and A130V, which, to summarise, fall in the functional domain of the protein, alter the protein’s sequence, are predicted deleterious by two bioinformatic algorithms, and are not found in the population. Therefore, these would be good candidates for functional follow-up.
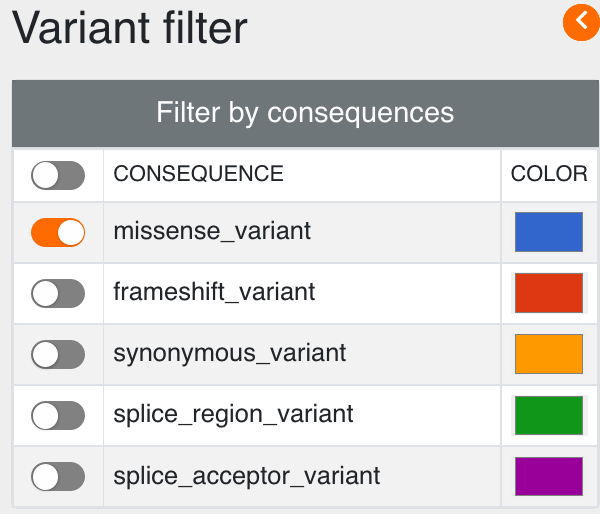

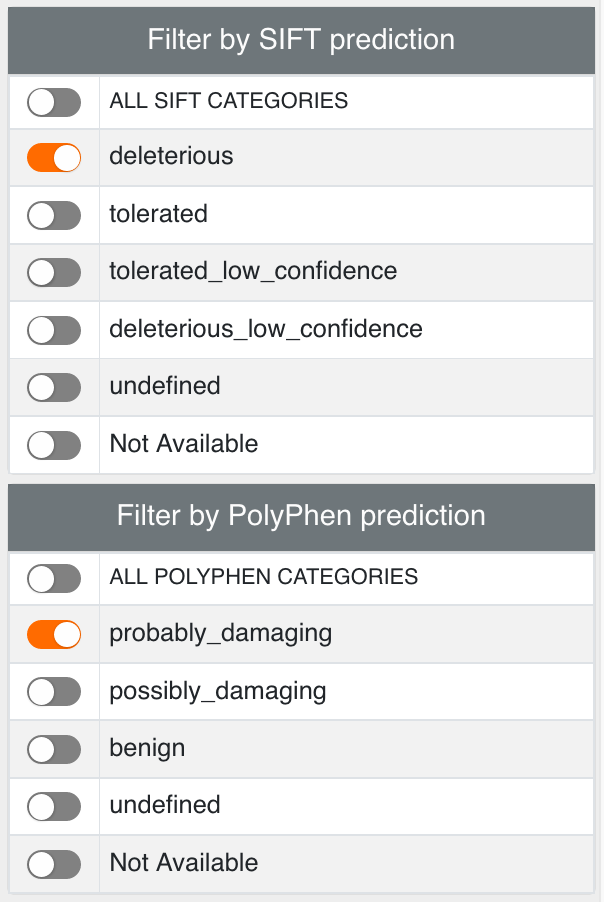


In O’Shea *et al* (2017), the missense mutants were functionally tested via deubiquitinase assays, and indeed S98R was found to have lost this function and therefore became a plausible melanoma-predisposing candidate variant, in addition to the frameshift and the splice acceptor variant.

However, this is not the only strategy we could follow in order to prioritise variants: there are many other criteria that we could apply to our variants, depending on our particular project set up. For example, we could also look at those protein-altering variants reported in the ClinVar database (R114H, K120E and R150C), although these have been previously described as present in the population and/or have conflicting pathogenicity predictions.

Prioritised variants after filtering steps:


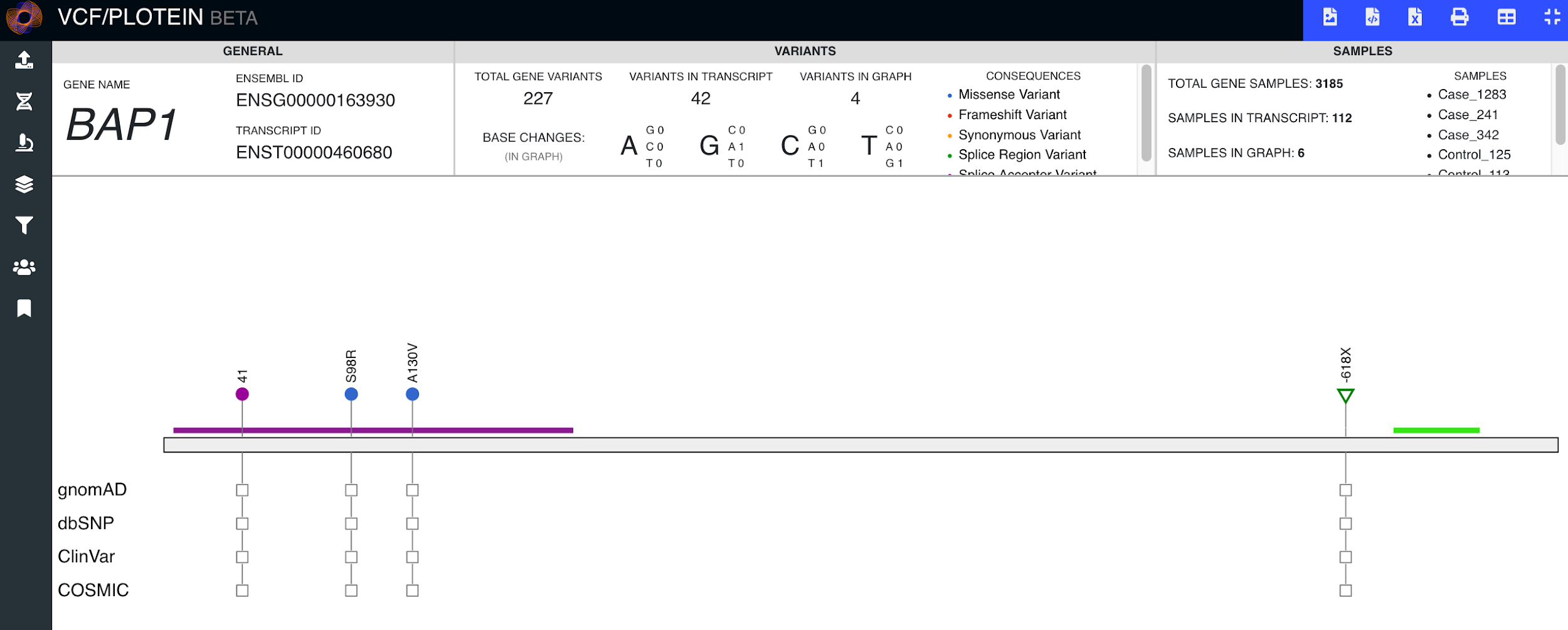

Supplement: btz458_Supplementary_Materials [file btz458_supplementary_materials.zip › btz458-suppl_data/Supplementary_text.docx]
