# Supplementary figures and images for: VCF/Plotein: visualization and prioritization of genomic variants from human exome sequencing projects
Source: Bioinformatics. 2019 Jun 4;35(22):4803–5. doi: 10.1093/bioinformatics/btz458 (PMC6853650; doi:10.1093/bioinformatics/btz458)

## Process flow

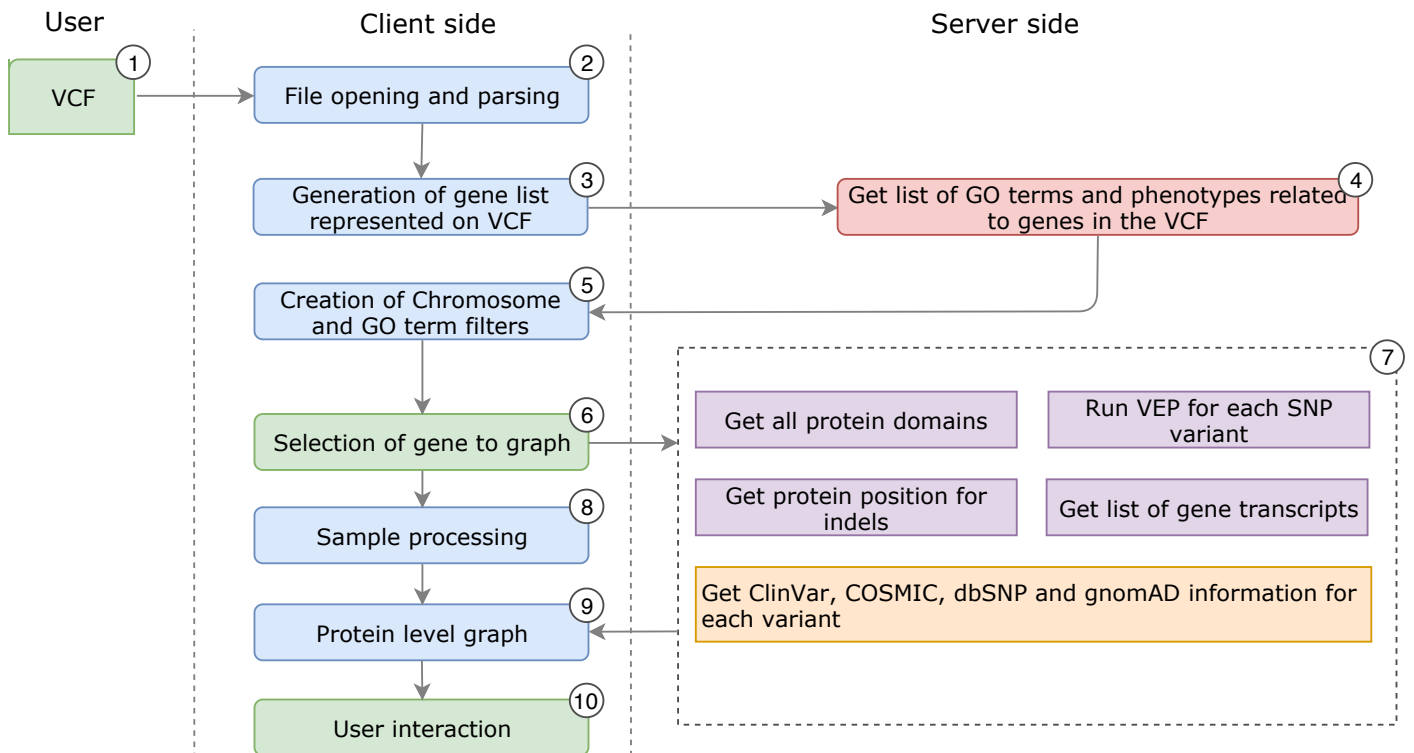

## System architecture

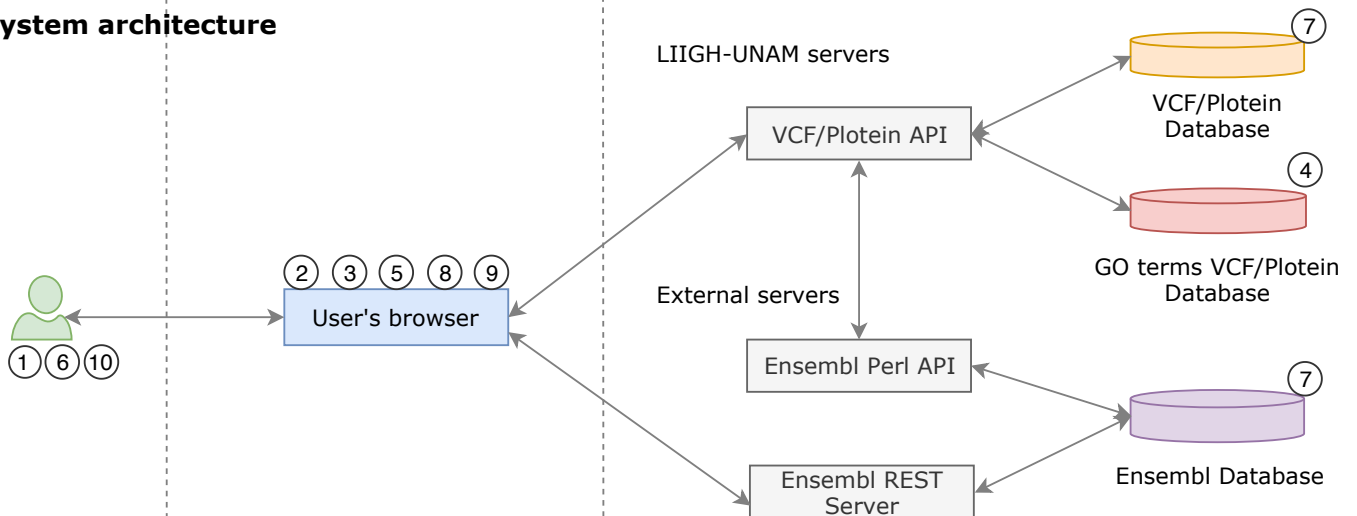

Supplement: btz458_Supplementary_Materials [file btz458_supplementary_materials.zip › btz458-suppl_data/Supplementary_Figure_1.pdf]

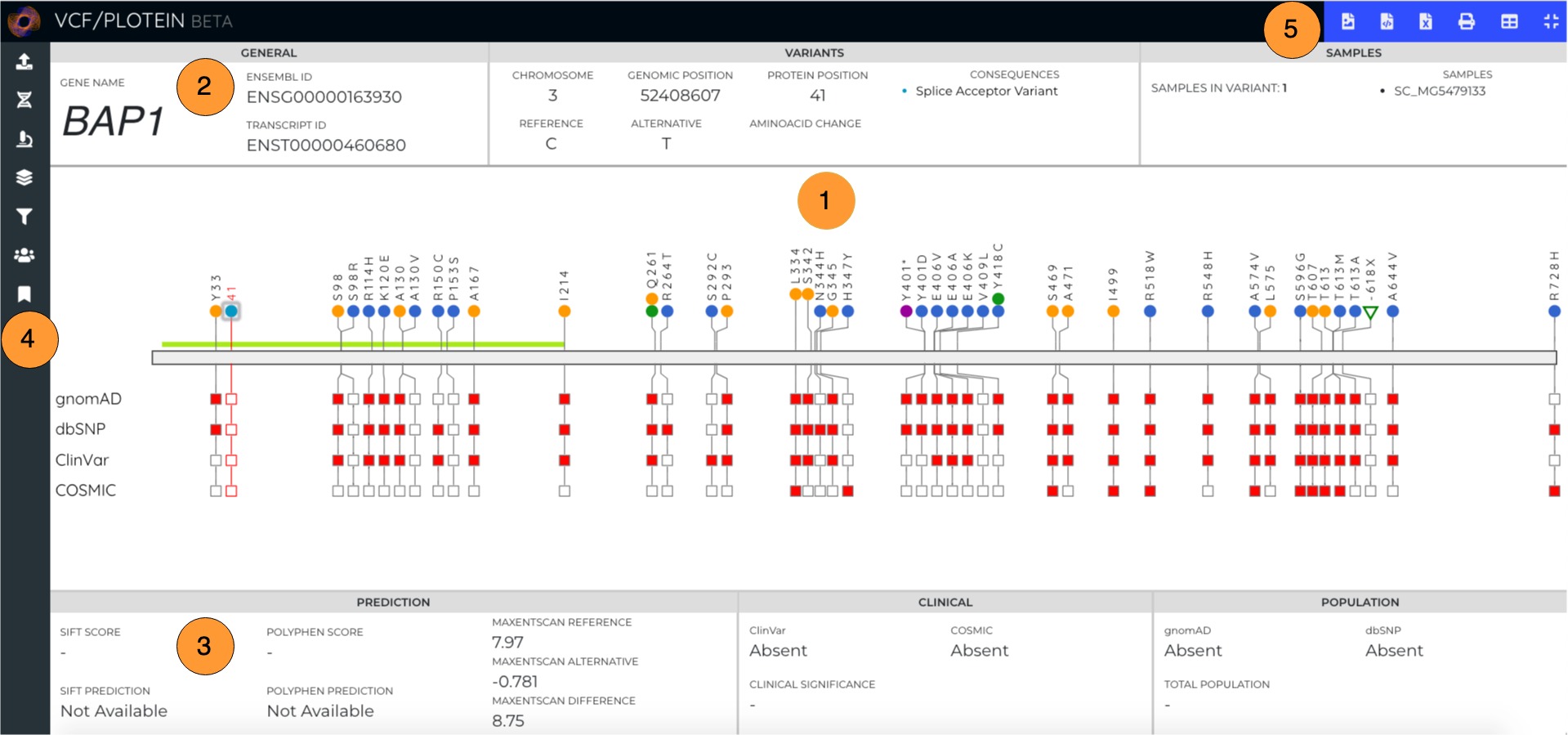

Supplement: btz458_Supplementary_Materials [file btz458_supplementary_materials.zip › btz458-suppl_data/Supplementary_Figure_2.jpg]

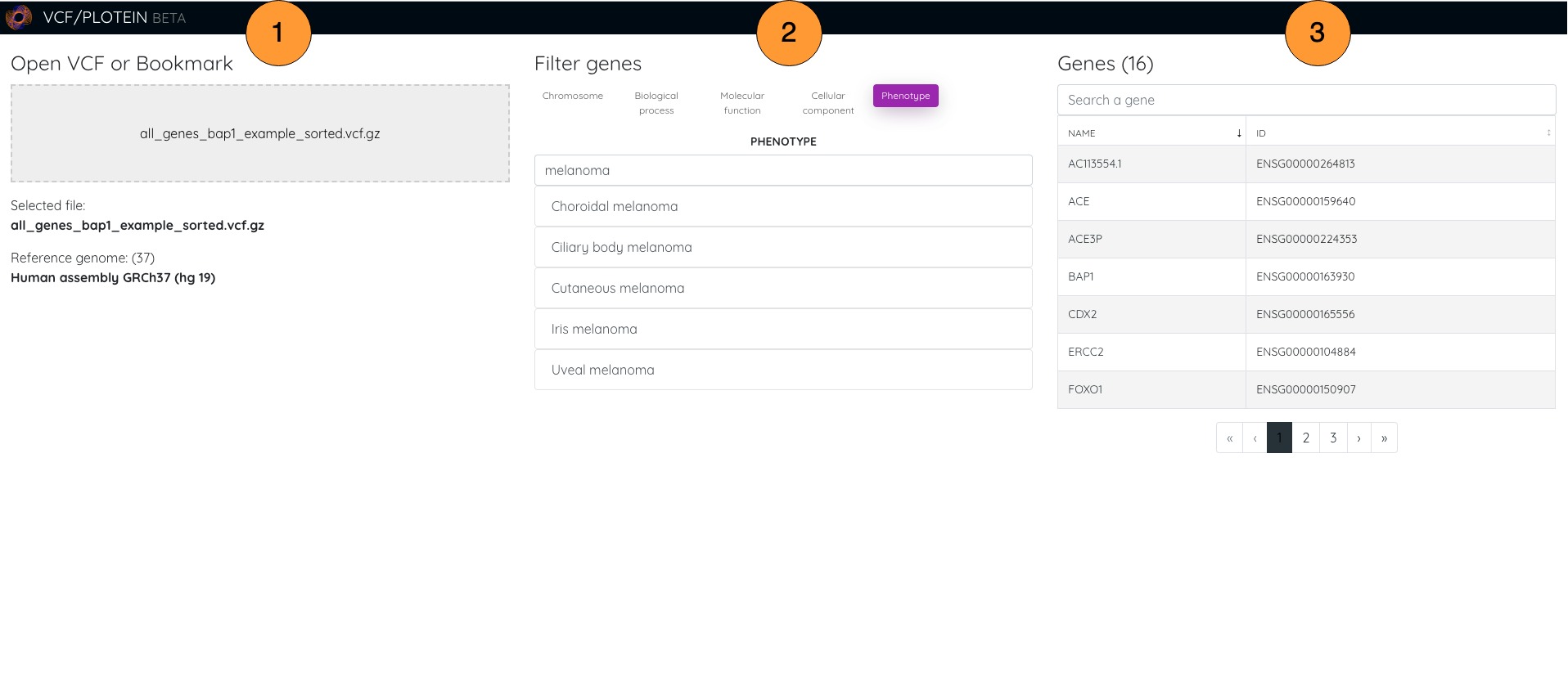

Supplement: btz458_Supplementary_Materials [file btz458_supplementary_materials.zip › btz458-suppl_data/Supplementary_Figure_3.jpg]
